# Amantadine in the Treatment of Sexual Inactivity in Schizophrenia Patients Taking Atypical Antipsychotics—The Pilot Case Series Study

**DOI:** 10.3390/ph14100947

**Published:** 2021-09-22

**Authors:** Marek Krzystanek, Anna Warchala, Beata Trędzbor, Ewa Martyniak, Katarzyna Skałacka, Artur Pałasz

**Affiliations:** 1 Clinic of Psychiatric Rehabilitation, Department of Psychiatry and Psychotherapy, Faculty of Medical Sciences in Katowice, Medical University of Silesia, 40-635 Katowice, Poland; awarchala@sum.edu.pl (A.W.); beataziarko@poczta.onet.pl (B.T.); emartyniak@sum.edu.pl (E.M.); 2 Institute of Psychology, University of Opole, 45-040 Opole, Poland; katarzyna.skalacka@uni.opole.pl; 3 Department of Histology, Faculty of Medical Sciences in Katowice, Medical University of Silesia, 40-752 Katowice, Poland; artiassone@gmail.com

**Keywords:** amantadine, sexual disfunctions, sex drive, libido, schizophrenia, hyperprolactinemia, antipsychotic drugs, atypical antipsychotics

## Abstract

Sexual dysfunctions in people with schizophrenia are more severe than in the general population and are an important element in the treatment of schizophrenia. The mechanism of sexual dysfunction in patients treated for schizophrenia may be related to the side effects of antipsychotic drugs (hyperprolactinemia, suppression of the reward system), but it may also be related to the pathogenesis of schizophrenia itself. The aim of the study was to present the possibility of using amantadine in the treatment of sexual dysfunction in schizophrenia without the concomitant hyperprolactinemia. In an open and naturalistic case series study, five men treated for schizophrenia in a stable mental state were described. All patients reported a prolonged lack of sexual desire and sexual activity prior to treatment with amantadine. After exclusion of hyperprolactinemia, patients received amantadine 100 mg in the evening. Sexual dysfunction was assessed using subscales of the 14-point Short Form of the Changes in Sexual Functioning Questionnaire (CSFQ-14). On subsequent visits after 1, 2 and 3 months of administration of amantadine, an improvement in sexual functioning was observed in all patients. Although this is only the preliminary report, amantadine may become a new indication for the treatment of sexual dysfunction in schizophrenia patients.

## 1. Introduction

Sexual dysfunctions in the schizophrenic population are more frequent and severe than in the healthy population [1,2]. For example, the prevalence of sexual dysfunctions in patients with schizophrenia treated with antipsychotics, depending on the study, concerns 44% [3], 50% [4] or 65% of patients [5], and even 82% in the group of men [6]. For these reasons, the treatment of sexual dysfunctions is a clinically important element in the comprehensive treatment of schizophrenia.

The pathogenesis of sexual dysfunction in schizophrenia is complex. They may be an adverse effect of antipsychotic drugs, but they may also be endogenous, related to the disease itself [7]. Antipsychotic drugs can cause sexual dysfunction by increasing the level of prolactin, on the other hand, the antidopaminergic mechanism of action of antipsychotic drugs itself may contribute to suppressing the reward system and reducing sexual desire. The mechanism of sexual dysfunction associated with the suppression of the mesocortical dopaminergic pathways is more likely in people treated with atypical antipsychotics, associated with a lower risk of hyperprolactinemia [8].

The routine therapeutic management in the treatment of sexual dysfunction in schizophrenia has so far included reducing the dose of the antipsychotic drug, switching to an atypical antipsychotic drug and psychotherapy, and in men adding a phosphodiesterase 5 inhibitor. Other substances that may have some efficacy in the treatment of sexual dysfunction in schizophrenia are bromocriptine [9,10], cabergoline [11], cyproheptadine [12], imipramine [13], shakuyaku-kanzo-to [14] and selegiline [15].

One of the therapeutic options in the treatment of sexual dysfunction in people with schizophrenia is also amantadine [16]. It has long been used in this indication for the treatment of sexual dysfunction associated with hyperprolactinemia side effects of antipsychotic medication [17]. Little is known about the possible use of the prodopaminergic effects of amantadine in patients treated for schizophrenia with sexual dysfunction without concomitant elevation of prolactin.

Atypical antipsychotics act as more or less selective antagonists of dopamine receptors in the limbic structures. Amantadine in turn distinctly facilitates dopamine signaling by elevation of neurotransmitter level in the mesolimbic pathways via inhibition of dopamine transporter1 (DAT1). It can stimulate DAT-controlling presynaptic D2 receptors of ventral tegmental area (VTA) neurons and act as an D1 receptor agonist of the spiny nucleus accumbens neurons [18]. Long-term treatment with amantadine co-administered with imipramine resulted in up-regulation of D2 and D3 receptors in the rat brain [19].

**Figure 1:**
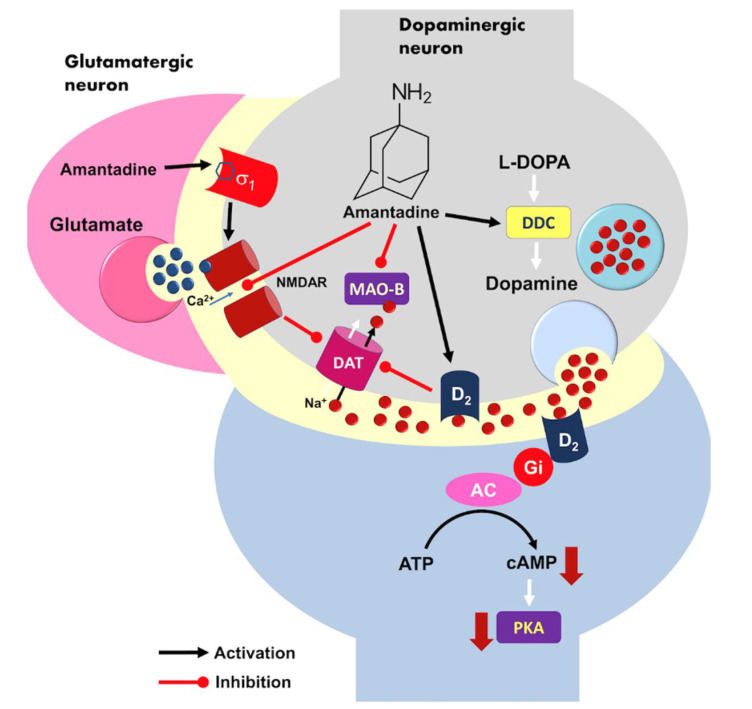
A model of possible molecular mechanism of amantadine action at the level of brain dopaminergic signaling. Activation of presynaptic dopamine receptor D2 may reduce dopamine transporter 1 (DAT1) activity in response to high neurotransmitter and elevates dopamine level within synaptic cleft. D2 is a Gi-coupled receptor, its excitation causes an inhibition of the adenylyl cyclase (AC) activity, decreased cAMP production and finally silencing of protein kinase A (PKA)-dependent neuronal signaling pathway. Amantadine is also able to inhibit monoaminoxidase B (MAO-B) but it does support L-DOPA decar-boxylase (DCC) activity in the presynaptic neuron. DDC—L-DOPA decarboxylase; MAO-B—monoaminoxidase B; DAT—dopamine transporter; AC—adenyl cyclase; PKA—protein kinase A.

Amantadine also increases the L-DOPA decarboxylase (DCC) action, but it blocks monoamine oxidase B (MAO-B) activity in the presynaptic dopaminergic neuron [20]. Acting as a weak noncompetitive N-methyl-D-aspartate receptor (NMDAR) antagonist, amantadine silences receptor ion current and finally inhibit glutamate signaling [21]. This follows DAT1 blockage and increase in local dopamine concentration that plays an important role in the origin of the pharmacological effect [22]. The mechanisms of amantadine are summarized in Figure 1.

So far, there is only one report in the medical literature on the possibility of improving primary sexual dysfunctions in schizophrenia with amantadine [16]. This problem has not been studied for 23 years. Since the problem of the lack of desire and other sexual dysfunctions is common and requires new pharmacological methods to help patients, the authors decided to describe a series of cases of male patients diagnosed with schizophrenia, who were treated with amantadine due to complete loss of sexual desire and activity.

## 2. Results

**Figure 2:**
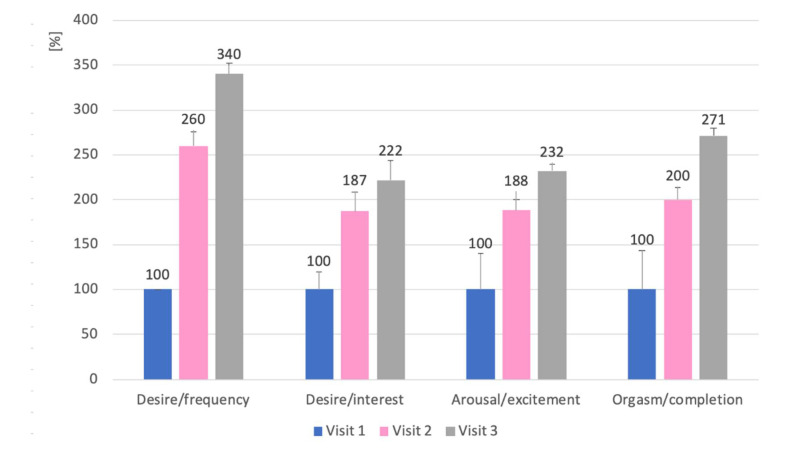
Change in mean values (+SD) of the CSFQ subscales at Visit 2 and 3 versus the values obtained by patients (*n* = 5) at Visit 1. Results are expressed as a percentage of the values at Visit 1 treated as 100%.

Adding amantadine to antipsychotic treatment significantly improved sexual function in all CSFQ subscales in all patients. The greatest improvement was observed in the Desire/frequency subscale—patients had an average score 2.4 times higher than at the beginning of the treatment (F(2,8) = 105.41; *p* < 0.001; eta^2^ = 0.963). After 3 months of administration of amantadine, the Orgasm/completion subscale was also 1.7 times better (F(2,8) = 41.75; *p* < 0.001; eta^2^ = 0.913). The improvement in the other subscales was similar—patients achieved mean scores 1.2–1.3 times higher than the baseline values (for Desire/interest subscale: F(2,8) = 60.88; *p* < 0.001; eta^2^ = 0.938; for Arousal/excitement subscale F(2,8) = 70.58; *p* < 0.001; eta^2^ = 0.946). Figure 2 shows the mean scores of all patients on the CSFQ subscales in relation to the values obtained at Visit 1, and Table 1 presents the statistical significance of improvement between the first and the last visit. Additionally, Table 2 presents the results in individual subscales of the patients’ CSFQ scale at subsequent visits.

As the patients indicated the time before falling asleep as the time of planned sexual activity, all received amantadine in the evening. No worsening of schizophrenia symptoms and no significant side effects were observed in any of the patients. Two patients experienced an increase in time to fall asleep, which resolved after changing the time of administration of amantadine. Individual case reports are summarized below.

### 2.1. Case 1

Case 1 is a 42-year-old patient who has been treated for schizophrenia for 20 years. In the past, he had irregular intake of antipsychotics and treatment intolerance was the reason for withdrawing his medications. In 2015, he was given aripiprazole 15 mg for the first time, which he tolerated well. Initially, he took the form of long-acting injection, but decided that the oral form was better for him. In 2017, he complained of sexual dysfunction and asked to be prescribed a phosphodiesterase 5 inhibitor. In the interview, it turned out that the patient rarely thought about sex and did not want to engage in sexual activity, and that he did not (or did not notice) any erections not even related to sexual activity. The desire disorder had occurred before with treatment with other antipsychotics. When the patient stopped taking his previous antipsychotic drug and before starting aripiprazole, he was not sexually disturbed. The patient’s serum prolactin was within the normal range. The patient was offered to administer amantadine 100 mg in the evening. He returned after a month with improvement, reporting he thought about sex at least twice a week, began to feel pleasure and interest in watching short pornographic films, and that he began to feel little pleasure in having sexual fantasies. He became sexually active twice, in one case he easily obtained an erection and kept it until the end of intercourse. After another month, the patient reported that he thought about sex several times a week, continued to enjoy pornographic material, tried to engage in sexual activity at least twice a week, and in half of the cases he easily obtainobtains an erection and maintains it until the end of intercourse. The patient did not report problems with ejaculation and orgasm when he had full intercourse, and the quality of his orgasm increased with treatment. The results of each subscale of the CSFQ-14 are presented in Figure 2. There was no worsening of psychotic symptoms and sleep disturbances in the patient. The tolerance of amantadine was good.

### 2.2. Case 2

Case 2 is a 47-year-old patient who has been treated for schizophrenia for 15 years. He has been taking quetiapine 600 mg as prolonged-release tablets since 2012. During his visit in 2019, he mentioned that he generally felt sexually cold and that he had very rare relations with his wife, despite the fact that they are in a very close romantic relationship. The sexology interview revealed that the patient thought about sex very sporadically—about once a month, did not feel the desire to engage in sexual activity and had no erections apart from morning erections, but notices it on average once a month. The serum prolactin level was within the normal range. He was given 100 mg of amantadine which he was supposed to take in the evenings. On the next visit after a month, the patient stated that he thinks more about sex, about once a week wants to engage in sexual activity, and he had intercourse twice, in which he easily obtained an erection once that lasted throughout the intercourse. After 5 weeks, at the next visit, the patient reported an improvement in sexual activity. He fantasized about sex several times a week, had sexual intercourse (sometimes masturbation) 1–2 times a week, during which he was able to easily obtainobtain and maintain an erection. The patient claimed that every time he had an erection and managed to end the intercourse, he would ejaculate. On each subsequent visit, he reported an improvement in the pleasure associated with orgasm. The results of each subscale of the CSFQ-14 are presented in Figure 2. The patient tolerated amantadine well and did not report any adverse effects.

### 2.3. Case 3

Case 3 is a 33-year-old patient who has been treated for schizophrenia for 10 years. He takes aripiprazole at a dose of 15 mg and clozapine at a dose of 300 mg. He has been in stable remission for many years. In 2020, during a visit, he reported that he had met a girl but, even though he was attracted to her, he did not feel sexual desire towards her. He claimed that for several months he had not felt the desire to have sex and had not masturbated. He rarely fantasized sexually, tried to watch pornographic material sometimes, but did not enjoy it. He had erections in the morning several times a month, but was unable to obtain an erection on his own. Serum prolactin was within the normal range. He was offered to add amantadine 100 mg in the evening to his antipsychotic. After 5 weeks, on the next visit, he said that he had a much greater desire for sex and is trying to initiate sexual activity at least once a week. The frequency of fantasizing about sex did not increase, but he began to search for and enjoy watching pornographic material on the Internet. He had more frequent morning erections and with his partner he was able to obtain an erection more easily, which lasted during intercourse in half of the cases to the end. He was pleased with the orgasm. After 4 weeks, he stated that he was satisfied with the improvement, fantasized about sex more often and was sexually active more than twice a week, he enjoyed both viewing pornography and orgasm, he usually managed to obtain an erection and keep it until the end of intercourse. He did not complain about problems with ejaculation. The results of the individual CSFQ-14 subscales are presented in Figure 2. Due to occasional problems with falling asleep (he fell asleep 1–2 times a week 1–2 h later than before taking amantadine), the dose of amantadine was shifted to 4:00 p.m. On the third visit, he did not complain about any problems with falling asleep. In addition, the patient did not report any side effects of amantadine.

### 2.4. Case 4

Case 4 is a 31-year-old patient who has been treated for schizophrenia since 2013. He was in stable remission since 2016, when treated with aripiprazole 15 mg daily. Previously, he was taking medication irregularly. In 2018, he asked to talk about his sexuality. He admitted that he wanted to start a sex life with his girlfriend, but that he had not had a partner before. He admitted that despite a strong emotional relationship, he did not feel any desire towards his partner and did not feel sexual arousal. He had erections in the morning at least once a month. He rarely had sexual fantasies and did not watch pornographic material because he felt no interest. Serum prolactin level was within the normal range. He was offered to add amantadine 100 mg in the evening to his treatment. At the next visit after 5 weeks, he reported a change in his sexual activity. He spontaneously fantasized more often about sex with his partner, it gave him little pleasure. He attempted intercourse twice, the first time he ended the intercourse with an ejaculation, the second time he failed to obtain a sufficient erection. After a month, he reported an increase in the number of times he had intercourse; in most cases, he had no problems with obtaining and maintaining an erection and the intercourse used to end with ejaculation. He felt a little more pleasure in sex in general and a lot of pleasure in having an orgasm. The results of the individual CSFQ-14 subscales are shown in Figure 2. The patient tolerated amantadine well and did not report any adverse events.

### 2.5. Case 5

Case 5 is a 64-year-old patient who has been treated for schizophrenia from the age of 19. Since 2014, he has been in a stable remission on risperidone, initially 6 mg daily, and since 2018 on risperidone in the form of a long-acting injection 50 mg i.m. every 2 weeks. In 2019, he separated from his wife and met a new partner. At the visit, he said his sexuality “didn’t work.” Despite frequent thoughts about sex, he did not feel any desire to have intercourse and, despite attempts to break through, he did not have sex with the partner, despite the fact that they had known each other for over 3 months. He had occasional erections in the morning, but failed to obtain an erection either by viewing pornographic material or masturbating. In the serum prolactin test, the result was at the upper limit of the normal range (18 ng/mL). It has been proposed to add amantadine 100 mg in the evening to the treatment. After a month he came forward smiling and said that “something started”. Spontaneous sexual fantasies occurred, and he enjoyed browsing pornographic material on the Internet. He had intercourse about once a week, obtained an erection easily in half of the cases, and ended intercourse with a satisfying orgasm in half of the cases. He reported even greater improvement after the next month. He found it was easier to obtain an erection, he fantasized sexually every day, had more sexual intercourse, and had greater sexual desire. The results of the individual CSFQ-14 subscales are shown in Figure 2. At the second visit, the patient reported that he fell asleep almost every day 2–3 h later than before. The time to take amantadine was moved to 4 p.m. At visit 3, he reported occasional problems falling asleep—about once a week he fell asleep 1–2 h later than usual. He did not report any other adverse effects related to the initiation or continuation of treatment with amantadine.

## 3. Discussion

The described study is the first clinical report in two decades concerning the possibility of treating drug-induced sexual disorders in schizophrenia with amantadine. A study by Valevsky et al., was, similar to our study, a short research report conducted on a small group of twelve men suffering from schizophrenia [16]. It was shown that amantadine administered for 6 weeks causes improvement in the field of desire, erection and sexual satisfaction. These results are consistent with the results of our report; however, the results presented now cover a longer period of observation.

In the described 3-month observation period, a constant improvement in sexual function was observed, the largest in terms of desire and orgasm. This suggests that people with schizophrenia treated with antipsychotic drugs can achieve greater improvements in sexual function along with the duration of treatment with amantadine. The shorter follow-up period in the study by Valevsky et al., may explain why no changes in ejaculatory function were observed in those patients—perhaps they would have occurred, as in this case series study after a longer treatment period.

**Figure 3:**
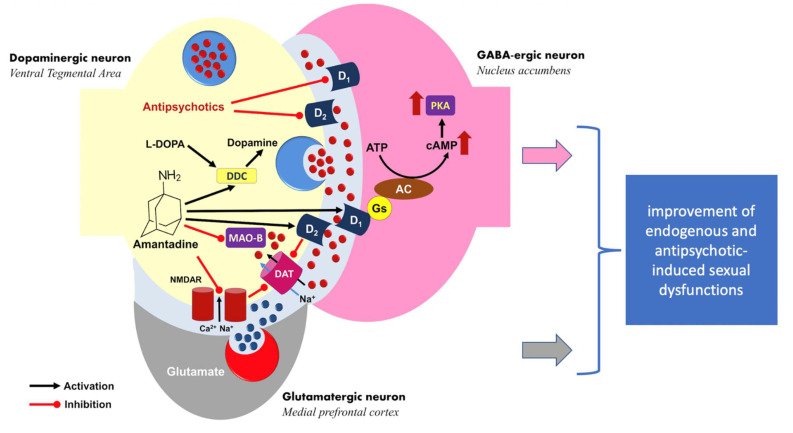
Hypothetical model of the mechanism of sexual functioning improvement by amantadine. The drug acts as a prodopaminergic modulator of the reward system (ventral tegmental area and nucleus accumbens), increasing the dopaminergic activation suppressed by antipsychotic therapy. DDC—L-DOPA decarboxylase; MAO-B—monoaminoxidase B; DAT—dopamine transporter; AC—adenyl cyclase; PKA—protein kinase A.

In a study by Valevsky et al., no prolactin test was performed. It is, therefore, possible that the sexual dysfunctions observed at that time were caused by hyperprolactinemia. In this context, the case series study described here may be the first report on effective amantadine treatment for sexual problems in people with schizophrenia in whom sexual dysfunction is related to the suppression of the reward system by an antipsychotic drug. In such a model of the development of antipsychotic-drug-induced sexual dysfunctions, amantadine could play the role of a modulator, increasing dopaminergic firing in the structures of the reward system in the mesolimbic system, on which antidopaminergic drugs are active (Figure 3).

The presented case series does not prove that the mechanism of action of amantadine is as outlined above. The case series study was only a naturalistic study and was carried out without placebo control. For this reason, the results obtained by us should be interpreted with great caution as preliminary and requiring confirmation in a placebo study. On the other hand, amantadine in the described group of patients showed a spectacular effect, improving sexual dysfunctions in people with schizophrenia, whose sexual functioning was suppressed. For this reason, the demonstrated efficacy of treating sexual dysfunction with amantadine in patients with schizophrenia is a clinical rationale for research into a new indication for amantadine in the treatment of sexual dysfunctions in schizophrenia.

Sexual disturbances may also be an immanent feature of schizophrenia, unrelated to the action of antipsychotic drugs [7]. This is indicated by the results of the study by Dembler-Stamm et al. [2], in which, using the Derogatis Inventory for Sexual Function (DISF), a greater severity of sexual disorders was shown in four of the five DISF subscales: “sexual cognition and fantasy arousal,” “sexual behavior and experience,” “orgasm,” and “sexual drive and relationship.” The limitation of this study was, as in our study, the small number of respondents (*n* = 19, men = 17). The sexual dysfunction profile in the study by Dembler-Stamm et al., resembles the group of patients we described, but all of them were also taking atypical medication or antipsychotics.

From this perspective, in patients treated for schizophrenia, endogenous sexual disorders and drug-induced disorders may overlap, and we have probably studied this group of patients. Amantadine, thanks to its mechanism of action, can correct both ways of sexual dysfunction, but to confirm this model, it would also be necessary to examine the effect of amantadine on sexual dysfunction in untreated patients with schizophrenia. This research problem may also be the subject of further research.

It is known that atypical antipsychotics differ in the incidence of sexual dysfunction [1]. Aripiprazole, administered as monotherapy in two of the patients described in our study, and quetiapine (Case 2) are among those antipsychotic drugs associated with the lowest risk of sexual dysfunction. However, as the described group shows, in selected patients, these disorders may be of significant intensity regardless of the type of antipsychotic. Therefore, in addition to considering the objective medical data from meta-analyzes when choosing a drug, a personalized approach is also needed [23] and in a situation where sexual dysfunctions occur even after the drug with a low risk of causing these problems, it is necessary to look for a solution to the patient’s problem, for example in the form of amantadine adding.

It is worth emphasizing the good tolerability of amantadine 100 mg in the described patients. This is especially meaningful in the context of potential concerns about the risk of the psychodysleptic effect of prodopaminergic drugs in schizophrenia. No worsening of schizophrenia symptoms was observed in any of the patients.

The limitation of the described study, apart from the number of patients, is also the gender of the patients—it only concerns men. Sexual dysfunctions in schizophrenia affect both women and men equally [7]; therefore, a study a similar study among women diagnosed with schizophrenia should be conducted.

## 4. Materials and Methods

The study covered five cases of patients chronically treated for paranoid schizophrenia in remission, diagnosed on the basis of ICD-10 research criteria. The criteria for selecting the described cases into a series of cases were the lack of desire and sexual activity despite having a sexual partner, lasting at least 3 months, and the absence of hyperprolactinemia. All patients were in stable remission of schizophrenia for at least 6 months before starting treatment with amantadine. None of the patients underwent medical treatment due to accompanying physical illness. Plasma prolactin level testing was a routine diagnostic test to differentiate the cause of sexual dysfunction. Blood samples for laboratory testing of serum prolactin levels was drawn between 7–8 am on an empty stomach. Laboratory norm of prolactin for men was <20 ng/mL.

The sexual functioning and mental state were assessed every 4 weeks ± 1 week. Before starting amantadine, the mechanism of action of amantadine was explained to each patient and information on potential side effects was provided. Each patient consented to off-label treatment with amantadine.

Patients received amantadine 100 mg per day at least 1 h before the scheduled time of sexual activity. The patients were informed that in the event of significant side effects or worsening of schizophrenia symptoms, they should discontinue amantadine and contact their physician. In addition to amantadine, patients continued the existing antipsychotic drug as the basic treatment for schizophrenia.

A 14-point Short Form of the Changes in Sexual Functioning Questionnaire (CSFQ-14) [24,25] was used to assess sexual functions. As the patients did not report sexual activity prior to the administration of amantadine, the CSFQ subscales were used for the evaluation, which allows us to precisely assess which sexual functions are disturbed and their possible changes during amantadine treatment. Subscale scores were calculated by summing the values of selected CSFQ items: Desire/frequency subscale—items 2 + 3, Desire/interest subscale—4 + 5 + 6, Arousal/excitement subscale—7 + 8 + 9 and Orgasm/completion subscale—items 11 + 12 + 13. Before collecting the completed CSFQ, the doctor checked each time whether the patient had answered all the questions in the questionnaire.

The statistical analysis of obtained results was performed with the Student’s *t*-Test for paired data (for difference between the first and the last visit) and the nonparametric Friedman Test for repeated measures (for general effect). Descriptive statistics include mean values, confidence intervals for the subscales, and the amount of improvement expressed as a percentage.

## 5. Conclusions

Amantadine may become a new indication for the treatment of sexual dysfunction in schizophrenia without concomitant hyperprolactinemia in patients who take antipsychotic drugs. Due to the limitations of the study, this conclusion has preliminary character and needs to be validated in controlled clinical trials in a larger patient population.

## 6. Patients

The retrospective case reports were used to prepare the publication. The data came from the outpatient clinic of one of the authors (M.K.). Patient data were anonymized for this report, making it impossible to identify them. The patients were informed about the mechanism of action for amantadine, potential side effects and the off-label use before taking the drug. Before the treatment, the patient consented to add-on treatment with amantadine.

## Figures and Tables

**Table 1:** Significance of the differences between the first and the last visit in CSFQ subscales.

Title	Δ Mean	SD	95%CI	t	df	*p*	d
Desire/frequency	−4.80	0.84	(−0.84, −3.76)	−12.83	4	0.001	0.84
Desire/interest	−5.60	1.52	(−7.48, −3.72)	−8.26	4	0.001	1.52
Arousal/excitement	−6.60	1.52	(−8.48, 4.72)	−9.73	4	0.001	1.52
Orgasm/completion	−7.20	2.05	(−9.74, −4.66)	−7.86	4	0.001	2.05

Note: Δ Mean—difference in means between the last and the first visit; SD—standard deviation; 95%CI—95% confidence interval; t—Student’s *t*-Test for paired data; df—degree of freedom; p—significance; d—Cohen’s effect size.

**Table 2:** Results in individual subscales of the patients’ CSFQ scale at subsequent visits.

Visit No	SCFQ	Patient 1	Patient 2	Patient 3	Patient 4	Patient 5	Mean [95%CI]
Visit 1	Desire/frequency	2	2	2	2	2	2.0 [2.0, 2.0]
	Desire/interest	4	4	5	4	6	4.6 [4.0, 5.4]
	Arousal/excitement	3	4	8	4	6	5.0 [3.6, 6.6]
	Orgasm/completion	3	3	7	3	5	4.2 [3.0, 5.8]
Visit 2	Desire/frequency	5	6	6	5	4	5.2 [4.6, 5.8]
	Desire/interest	8	9	9	6	11	8.6 [7.0, 9.8]
	Arousal/excitement	9	8	11	9	10	9.4 [8.6, 10.4]
	Orgasm/completion	8	7	9	8	10	8.4 [7.6, 9.4]
Visit 3	Desire/frequency	7	7	8	6	6	6.8 [6.2, 7.4]
	Desire/interest	10	10	11	7	13	10.2 [8.4, 11.8]
	Arousal/excitement	11	12	13	11	11	11.6 [11.0, 12.4]
	Orgasm/completion	11	12	12	12	10	11.4 [10.6, 12.0]

Note: 95%CI—95% confidence interval.

## Data Availability

Data is contained within the article.
